# Locus of Control and Self-Directed Learning Readiness of Nursing Students during the COVID-19 Pandemic: A Cross-Sectional Study from Saudi Arabia

**DOI:** 10.3390/nursrep13040137

**Published:** 2023-11-30

**Authors:** Hanan A. Alkorashy, Hanan A. Alotaibi

**Affiliations:** 1 Nursing Administration & Education Department, College of Nursing, King Saud University, Riyadh 11362, Saudi Arabia; 2 Nursing Administration Department, Faculty of Nursing, Alexandria University, Alexandria 21526, Egypt; 3 Maternal and Child Health Nursing Department, College of Nursing, King Saud University, Riyadh 11362, Saudi Arabia; halotaibib@ksu.edu.sa

**Keywords:** locus of control, self-directed learning readiness, nursing students, Saudi Arabia, undergraduates, COVID-19 pandemic

## Abstract

Background: Coronavirus disease (COVID-19) has caused one of the worst global pandemics in recent decades. It has disrupted education systems worldwide, leading to a forced shift from traditional face-to-face to blended or fully distanced learning, requiring a higher level of student readiness for self-directed learning (SDL) and a more internal locus of control (LOC). Objective: This study explored the relationship between locus of control and level of readiness for SDL among Saudi nursing students and whether the COVID-19 pandemic has impacted this relationship. Methods: A cross-sectional correlational descriptive study was conducted to survey 277 Saudi nursing students enrolled in the bachelor program at one of the reputable universities in Saudi Arabia. An E-questionnaire containing two scales, the Self-Directed Learning Readiness Scale for Nursing Education, and the Locus of Control Scale, was used to collect data in addition to the selected participants’ characteristics. Results: Nursing students had a moderate-to-low level of readiness for SDL (mean = 144.0), and the majority had an external LOC. There was a significant association between locus of control and level of readiness for self-directed learning (r = 0.19 *, *p* = 0.001), and the internal locus of control was more significantly associated with self-directed learning (r = 0.22 *, *p* = 0.0001) than with external locus of control. Conclusion: The study findings indicate a propensity of respondents indicating an external locus of control, whereas most of the respondents’ reported levels of readiness ranged between low and moderate across all dimensions of self-directed learning. This study was not registered.

## 1. Introduction

As a result of the global Coronavirus disease (COVID-19) pandemic, education has undergone dramatic changes, including in Saudi Arabia. Following COVID-19, academic institutions were forced to shift their efforts to facilitate an abrupt and unexpected transition to online education and assessment [1]. Due to these efforts, e-learning has grown significantly, and nursing education is also now available via digital platforms [2]. The abrupt closure of educational institutions negatively affected students’ academic performance and achievement [3,4]. Additionally, it had an adverse effect on university students’ lives in all areas [5,6], including nursing students. Students’ educational requirements have been affected by heightened stress and anxiety due to COVID-19 [3,7,8]. Saudi Arabian universities had to take immediate action to contain the Coronavirus’ spread after unscheduled closures, which began on 9 March 2020.

Similar to other universities around the world, the College of Nursing at the targeted Saudi university was urged to develop online courses with reformatted content and innovative teaching methods within a relatively short timeframe in order to remain active during the COVID-19 pandemic while following preventative protocols and measures [6,7,8,9]. Nursing education has evolved from a traditional face-to-face model to one based on virtual learning modes [10]. Virtual education has necessitated the rapid conversion of in-person content into an online format, resulting in a lack of clinical practice opportunities for students, as is usual for traditional nursing programs [6]. Students had to adjust quickly to the challenges associated with virtual classes and assessments as a result of innovative approaches to assessment in this mode of education.

It is therefore necessary for students to consider new methodologies for organizing, preparing, and interacting with their studies in this context. As a result, students became more independent and self-directed when it came to completing course requirements. In online environments, self-directed learning is one of the best predictors of better learning outcomes and academic achievement [11]. A significant amount of research has shown that locus of control (LOC) and self-directed learning (SDL) have significant effects on students’ performance and readiness for online learning [11,12,13,14].

The locus of control refers to how one believes he or she can control oneself [13], while self-directed learning competency refers to the extent to which one accepts responsibility for learning. To be successful in the future, nursing students must develop their SDL to develop professionalism [15]. With distance learning, students manage a variety of circumstances to succeed academically while taking responsibility for their education [16]. Motivation to learn is derived from the LOC [17]. Global pandemics can negatively affect LOC and affect academic performance [18,19]. A lifelong learning process requires the acquisition of SDL and LOC, which enables individuals to critically evaluate the knowledge they have acquired [20,21]. COVID-19 presents similar challenges [6]. It has been found that integrating lifelong learning strategies into nursing education results in a higher level of education and professional competence [22], which in turn fosters the development of professional values and improves nursing outcomes [21,23]. There is a significant correlation between these traits and Saudi Vision 2030 [23,24,25]—a road map that seeks to transform health delivery systems, education, and nursing, among many other fields, in order to develop a globally competitive and prosperous country by 2030.

One of the major consequences of the COVID-19 pandemic has been psychological challenges [24]. Students of higher education have also been reported to experience stress and anxiety. During COVID-19, Saudi students were reported to experience moderate to extreme levels of anxiety, with stress levels perceived to be as high as 35% [25]. In addition, recent studies indicate that students’ perceived stress is significantly correlated with their locus of control [26,27] and that the locus of control influences their learning outcomes [28,29]. Concomitantly, a high level of academic achievement depends on their internal locus of control [29,30,31,32,33,34].

Developing SDL skills is crucial in preparing college students for life after graduation [33,35,36]. A study by Cheng et al. indicates that the learner formulates learning objectives, selects appropriate learning strategies, diagnoses learning needs, identifies resources, and evaluates learning outcomes, with or without external support [37]. SDL is utilized in a wide variety of contexts, including problem-solving, contract negotiations, distance learning, and clinical documentation [36]. As a result of SDL, nursing students develop independent learning skills [38]. Moreover, SDL enhances students’ self-confidence and motivation, which are vital to both their personal and professional success [39].

Furthermore, it fosters purposeful change, which is essential for effective personal and professional lives [22,40,41]. Lee et al. [22] found that SDL has an important direct impact on nursing students’ professional values. Considering the proficiency of the new generation in using the Internet and other information sources, the findings of this study should encourage nursing educators to promote SDL among nursing students. Researchers have consistently observed a strong correlation between SDL use and positive educational outcomes in various countries, including Oman, Saudi Arabia, China, and Turkey [30,32,38,39,42].

Assessing nursing students’ self-direction levels can be achieved through the measurement of their self-directed learning readiness (SDLR) [42]. A study conducted at Al-Jouf University in Saudi Arabia revealed that 77% of nursing students demonstrated high levels of SDLR [38], which positively correlated with academic performance in undergraduate nursing students [43]. Despite a study showing no significant gender-based differences in SDLR scores [44], Alsufyani et al. raised concerns about the involvement of factors such as gender, age, and clinical experience in SDLR scores [42].

The concept of LOC has been examined by Rotter in a psychological context [45], referring to individuals’ beliefs about their ability to control causality, situations, and life experiences. Among educators, LOC refers to the way students interpret the factors contributing to their academic success. It has been classified by Rotter [45] as internal or external. Individuals with an external LOC attribute their behavior to external influences, while internally oriented individuals believe that their behavior is primarily shaped by their own decisions and efforts. Moreover, LOC plays a critical role in motivating learning [46,47]. It is significantly linked to academic achievement [18], a crucial aspect for students [46]. According to past research, students with high internal LOCs are more likely to persist in online education and achieve higher academic outcomes than students with low internal LOCs [48,49,50]. By contrast, Harrell and Bower found no significant relationship between LOC and student persistence in online learning [51]. Bahçekapılı and Karaman concluded that external LOC negatively and insignificantly influences students’ academic achievement [52]. Even though students with high levels of internal LOC are better prepared for SDL in a traditional classroom setting than students with low levels of internal LOC, regardless of their year of study, Arkan et al. [15] analyzed the influence of internal LOC on nursing students.

Following numerous calls for an exploration of the impact of the COVID-19 pandemic across all sectors, particularly health and education, extensive research has been conducted to determine students’ readiness to embrace SDL [30,31,32,42,43,44]. While nursing students in Saudi Arabia experienced the COVID-19 pandemic, the literature is unclear as to whether the locus of control affects their SDL readiness level [33,34]. The current study fills this literature gap by exploring nursing students’ readiness for SDL and their locus of control. With many nursing schools using online learning platforms to guide student learning, this study’s potential contribution is more valuable than ever. Our study is the first to link students’ locus of control to their readiness to learn independently during COVID-19 outbreaks in Saudi Arabia. For nursing educators, academic leaders, educational psychologists, and policymakers, this study provides new insights into nursing students’ learning.

### 1.1. Aim of the Study

The purpose of this study was to investigate the relationship between LOC and readiness for SDL among nursing students during the first wave of the COVID-19 pandemic contingency.

### 1.2. Research Questions

1. What is the nature of the nursing students’ locus of control?
2. What is the level of readiness for self-directed learning among nursing students?
3. Is there a relationship between locus of control and readiness for self-directed learning among nursing students during the COVID-19 pandemic?

## 2. Methods

### 2.1. Study Design

For this study, a cross-sectional, descriptive, cross-sectional, correlational design was employed to examine relationships among the study variables, SDL and LOC. A cross-sectional study design involves the simultaneous collection and analysis of data for a given phenomenon. As well as describing a concept’s status and examining relationships and connections between variables, descriptive correlational studies do not infer causality [53].

### 2.2. Setting

During the academic year 2020–2021, this study was conducted at the Nursing College, at one of the reputable universities in Saudi Arabia. Currently, this Bachelor of Science in the Nursing program has eight levels, including classroom as well as laboratory activities that are integrated with clinical experiences. The study commenced during the first and the second academic semesters to include students who attended the classrooms, the nursing simulation labs, and/or practiced extracurricular activities.

### 2.3. Participants

Nursing students enrolled in a bachelor’s degree program at a Saudi Arabian university in the 2020–2021 academic year made up the study population (N = 967, 497 females and 470 males). To obtain a sufficient sample size, a convenience sampling methodology was used to select participants. Nursing students registered at the third through the eighth academic levels of their bachelor’s program who were available to participate in the survey at the time of data collection were invited. The sample size was calculated using Raosoft website’s sample calculator (http://www.raosoft.com/samplesize.html (accessed on 21 October 2023)). To achieve a medium effect size (f^2^ = 0.3), assuming a significance level (α) of 0.05 and a power of 0.95, a minimum sample size of 276 participants was required to detect the associations among the study variables. In order to account for attrition and/or withdrawals, an additional 5% of participants were invited, leaving 290 participants eligible to participate.

### 2.4. Eligibility Criteria

Nursing students (both males and females) enrolled at the third through eighth academic levels who were available during data collection and willing to participate in the study were eligible to participate in the study.

### 2.5. Measurements

Data collection was done using a structured self-report questionnaire, which consisted of three parts. The first part assessed participants’ demographic characteristics, including their age, marital status, academic level, permanent residence, and years of experience. The Self-Directed Learning Readiness Scale for Nursing Education, developed by Fisher et al. [36] and revised and validated by Fisher and King [54], was used in the second part of the study. The original version of this scale was created to help nursing educators diagnose the necessary attitudes, abilities, and personality characteristics for nursing students’ self-directed learning. There are 40 items on the scale, divided into three categories: self-management (13), learning desire (12), and self-control (15). To measure students’ responses, a Likert scale from 1 to 5 was used. For statements that were negatively stated, reverse scoring was implemented (e.g., strongly agree to strongly disagree). Overall scores ranged from 40 to 200, with higher scores reflecting stronger SDL readiness. Various nursing education studies [36,54,55,56] tested the validity and reliability of the scale, finding Cronbach’s alpha values between 0.70 and 0.85. The third part of the questionnaire used the Locus of Control Scale, developed by Dag [57], which was adapted from Rotter’s Internal-External Locus of Control Scale [45]. This scale aims to evaluate whether individuals believe that the consequences of their actions are internally or externally influenced. This scale includes 47 items, divided into five categories. They address a range of factors, namely personal control (18 items), belief in chance (11 items), meaninglessness of effort (10 items), fate (3 items), and an unjust world (5 items). The Likert scale was used to rank responses (1 = not at all suitable to 5 = fully suitable). Higher scores indicated a stronger belief in external LOC. Cronbach’s alpha and Pearson’s product-moment correlation test values of 0.88 and 0.92, respectively, were obtained for the original scale, indicating good internal consistency [57]. According to Beaton et al.’s guidelines for cross-cultural adaptation of self-report measures [58], the Locus of Control Scale was cross-culturally and linguistically adapted. Two bilingual nursing professionals independently translated the scale into English and conducted a blind back-translation into Turkish to determine construct validity. Three academics and two professionals proficient in both Turkish and English reviewed the scale, comparing the back-translations to the original. An online survey was conducted using Google Forms. Ten students participated in a pilot study to assess whether the scales were linguistically clear and culturally coherent in relation to the Saudi Nursing Academic culture and nursing practices. Since the students reported no problems with the clarity or relevance of the questionnaire, the pilot test responses were included in the main study. Cronbach’s coefficients for both scales produced a Cronbach’s ratio of 0.91 for the locus of control survey and 0.86 for the self-directed learning survey. The CHERRIES Checklist for electronic surveys was followed [59].

### 2.6. Data Collection Procedure

In accordance with COVID-19 restrictions and the university’s epidemic prevention and control policies, and after obtaining approval from the Standing Committee for Scientific Research Ethics at the university, all students were approached online, as physical contact was not possible. Nursing students who consented to participate were emailed a link to the online questionnaire by the college’s scientific research unit in coordination with researchers and the college’s academic advising committee. Academic Coordinators were given e-survey links to share with students via their academic emails. Data collection took place over a 12-week period from January to March 2021.

### 2.7. Ethical Considerations

Study approval was obtained from the Institutional Review Board (bioethical committee of the researchers’ university) (IRB log number KSU-HE-21-02. Furthermore, approval was obtained from the vice deanship for academic affairs, academic advisors, academic level coordinators, and faculty teaching nursing students. The authors gave their permission to translate the LOC scale and adapt both LOC and RSDL. As part of the consent process, participants were asked to click “agree” to confirm that they understood the purpose, nature, benefits, and uses of the data, as well as their voluntary acceptance of participation in the study. No names or personally identifiable information were collected in survey responses as a means of ensuring anonymity, indicating that the survey did not use the respondent’s IP address, username, contact information (e.g., email address), or respondent tracking functionality, and anyone with access to the survey could not relate a response to a respondent [59].

### 2.8. Statistical Analysis

A statistical analysis was performed with SPSS version 24 (IBM Corp., Armonk, NY, USA). The Excel spreadsheet was screened for missing and incomplete responses using data-cleaning techniques before being declared valid. Continuous quantitative variables were described after assessing their normal distributions, which were assessed using the Shapiro–Wilk test. Means and standard deviations (SD) of normal distribution variables were calculated, while frequencies (f) and percentages (%) were used to describe nominal categorical variables.

A descriptive statistic was used to summarize participants’ demographic characteristics and to assess their levels of RSDL and LOC to answer the first and the second research questions, including frequencies, percentages, means, standard deviations, minimums, and maximums. The Pearson product–moment correlation analysis was used to determine the relationship between students’ LOC and their readiness for SDL to answer the third research question. The statistical significance threshold was set as (*p* < 0.05).

## 3. Results

### 3.1. Descriptive Analysis

Out of the 290 student participants, 277 completed the electronic survey, yielding a response rate of 95.5%. Table 1 presents an overview of the demographic characteristics of participants. Female students constituted 57% of the participants, whereas 43% were male. The mean reported age was 20.5 (±1.6) with the majority (98.6%) being single. Regarding academic level, 29.2% were at Level 6, 23.5% were at Level 7, 16.2% at Level 4, 14% at Level 3, and 10.8% at Level 5. Most participants were residents of Riyadh City and resided in their family homes (92.4%).

*Research Question (1): What Is the Nature of the Nursing Students’ Locus of Control?*

The locus of control consists of five subscales: personal control, belief in chance, meaninglessness of effort, belief in fate, and belief in an unjust world. Table 2 shows that participants reported higher mean scores for external LOC (X = 86.2, SD = 19.7) than for internal LOC (X = 56.38, SD = 1.45), indicating that participants in this study believed that external factors or forces such as luck would determine their outcomes. The findings revealed that a higher mean score was reported for personal control (X = 56.38, SD = 1.45), followed by belief in chance (X = 32.21, SD = 7.97), belief in meaningless effort (X = 29.37, SD = 7.61), belief in an unjust world (X = 14.76, SD = 3.79), and belief in fate (X = 9.87, SD = 2.61).

*Research Question (2): To What Extent Are Nursing Students Ready for Self-Directed Learning?*

SDL was assessed using three subscales: self-management, desire for learning, and self-control. The results showed that almost 60% of participants reported a low level of readiness for SDL (144 ± 0.49), while 40% reported a high level. For the subscales, the highest mean score was for self-control (X = 52.36, SD = 12.45), followed by desire for learning (X = 45.02, SD = 10.38), and self-management (X = 39.52, SD = 9.04) (Table 3).

### 3.2. Inferential Analysis

*Research Question (3): Is There a Relationship between LOC and Readiness for Self-Directed Learning among Nursing Students during the COVID-19 Pandemic?*

Correlation analysis demonstrated a significant association between the locus of control and level of readiness for SDL (r = 0.19 *, *p* = 0.001), with the internal locus of control showing a more substantial association with SDL (r = 0.22 *, *p* = 0.0001) than the external locus of control, which exhibited no statistically significant association (r = 0.10, *p* = 0.08). Moreover, the self-directed learning subscales displayed statistically significant correlations with all the locus of control subscales. Table 4 presents the results of the correlation analyses.

## 4. Discussion

The study represents the first attempt to evaluate nursing students’ LOC and RSDL in Saudi Arabia during the COVID-19 outbreak. As a critical component of problem-solving abilities, SDL significantly contributes to nursing students’ clinical competence [60]. This study examined the nature of LOC and readiness for SDL among Saudi nursing students during the initial wave of the COVID-19 pandemic. The association between LOC and SDL readiness was also explored. A major strength of this study is its pioneering use of Dag’s [57] English version of the Locus of Control Scale, which was cross-culturally adapted and validated for Saudi culture within the context of the COVID-19 pandemic contingency, resulting in its robustness.

The findings indicated that the study variables had a notable association. According to the current study, the student population’s internal locus of control significantly decreased during the COVID-19 pandemic, whereas the external locus of control increased. In similar COVID-19 situations, previous studies found that the external locus of control was more prevalent among university students than the internal locus of control. Those findings are consistent with those of Misamer et al. [61], Wali et al. [62], and Hammoud [63], who observed that most of their studies’ participants displayed a higher external LOC than internal LOC, and the LOC shifted substantially from internal to external during the initial COVID-19 outbreak. It is likely that students experienced heightened stress as a result of the challenging nature of the pandemic and the rapid changes associated with this. Those with an external LOC tend to react emotionally and withdraw from stressful situations (such as the COVID-19 pandemic) as compared to those with an internal LOC, who are better able to cope with stress and utilize problem-solving strategies to cope with its consequences [62].

According to the current study, approximately 60% of nursing students were not prepared for SDL. This finding is consistent with Ballad et al. [30], Nazarianpirdosti et al. [64], and Dogham et al. [65]. In contrast, Samarasooriya and colleagues [66] and Alsufyani et al. [42] concluded that students who completed bridging programs or who were registered nurses (RN) were significantly more likely to be ready for SDLs if they had prior clinical experience and self-reliance. Nazarianpirdosti et al. [64] concluded in a previous systematic review that SDL was insufficient in this context. Further, nursing students reported that they were prepared for SDL to a moderate extent.

In comparison to self-management and the desire to learn, self-control was the most influential subscale regarding SDLR readiness. Kaur et al. [67], Aljohani and Fadila [68], and Ballad et al. [30] also found that the majority of participating Indian, Saudi, and Omani students demonstrated high levels of self-control. In other words, nursing students are fully aware of and accountable for their learning processes. According to the current findings, nursing students are capable of managing their own conduct in pursuit of their ideals and goals, as well as effectively handling their learning within the online educational platform (LMS-Bb) available during the pandemic [69]. In contrast, students demonstrated fewer abilities, attitudes, and personality traits related to SDL.

A significant relationship was found between LOC and SDL readiness levels among student participants, particularly in regard to the internal locus of control, according to the current findings. It was found that this internal LOC is significantly related to SDLR and all its dimensions, including self-control, self-management, and learning desire. Several studies have identified the association between internal LOC and academic self-regulation (self-control), including those by Sidola et al. [70], Syahputra and Affandi [71], Javidkar et al. [72] and Arkan et al. [15].

Based on this study, students with external LOCs were found to be low or moderately ready for SDL, and their achievements were largely determined by external circumstances. According to this association, students with an external LOC lack control over their behaviors, emotions, and thoughts in pursuit of long-term goals. In particular, they have difficulty managing their emotions, thoughts, behaviors, and energy in ways conducive to their academic achievement, well-being, and learning [70].

Referring to the findings of this study, the internal LOC was significantly positively associated with self-management and the desire to learn subscales. According to Rafique et al. [73], students who were internally controlled had a greater desire to learn as compared to those with an external LOC in terms of readiness for SDL. Students with an internal LOC showed greater confidence in executing their study plans, requesting timely assistance, managing their time, and setting learning goals, as well as having higher learning expectations. They also demonstrated effective self-management and a genuine interest in learning, as well as demonstrating more innovation, motivation, and sharing their ideas with colleagues and teachers. Externally controlled students, however, did not possess these characteristics and relied more on external support to attain their goals [73].

### 4.1. Limitations

As the study was conducted at a single nursing college, convenience sampling was used, and the sample size was relatively small, the findings cannot be generalized. The data were collected using an e-questionnaire that measured independent and dependent variables simultaneously. Therefore, it could not provide sound information regarding the causal relationships among the investigated variables. Also, this study focused on the relationship between LOC and SDLR and did not consider external variables that could affect students’ SDL, such as mood, health status, or gender. Therefore, further longitudinal studies are required. The findings also heavily depend on the COVID-19 crisis as the main precipitating factor and do not explore the causes of students’ external control and lack of SDL readiness.

### 4.2. Recommendations

The SDL process can be used to improve nursing students’ learning processes [22]. Nursing education has been shown to benefit from SDL as it has been significantly associated with academic achievement [74], professional competence, communication self-efficacy, assertiveness, accountability [64,75], and clinical competency [76]. The importance of SDL in nursing education should motivate nursing educators to encourage students to use SDL effectively.

Additionally, since SDL is a crucial component of nursing student clinical competence, it is necessary to encourage this form of education. Prior to incorporating SDL skills into their curriculum, nursing professors should train their students and impart these skills. Through the adoption of problem-based and student-centered curricula, nursing education and teaching methods can be improved. Considering that nursing students are not attaining the desired level of SDL, future studies should investigate factors influencing their readiness for SDL and evaluate the effectiveness of educational interventions. To understand the factors that facilitate and inhibit SDL, qualitative studies are necessary.

### 4.3. Further Studies

Future studies should replicate this study using different target settings and populations, including nursing and non-nursing health colleges. Although these findings are applicable at the target-setting (college) level, they can be replicated both nationally and internationally.

### 4.4. Conclusions

As indicated by the preceding findings, respondents had a tendency towards an external locus of control. Most respondents scored poorly across all dimensions of SDL readiness. Although the majority of respondents demonstrated low readiness levels for SDL, self-control is the most important dimension in comparison to self-management and desire to learn. According to this finding, nursing students can regulate their behavior and manage their learning effectively based on the available resources and their goals. A statistical analysis of the data indicated a significant positive correlation between internal locus of control and readiness for SDL. As a result, most student participants with an external LOC may benefit from educational administration and academic staff cultivating an environment where they feel confident that they will be able to execute their study plans, seek timely assistance, manage their time, set goals, and achieve higher learning expectations. They will be more innovative, motivated, inclined to share ideas with colleagues and teachers, skilled at self-management, and enthusiastic about learning.

Considering these findings, nursing students should be encouraged to develop a sense of self-direction so that they can become self-directed learners, a trait that is considered positive among nurses and known to enhance their ability to achieve their desired goals. In addition, the internal locus of control significantly contributes to all three dimensions of SDL readiness in nursing students. Nursing students’ internal locus of control is crucial to their developing lifelong learning strategies, becoming more competent in clinical situations, and succeeding in academic life.

### 4.5. Relevance to Clinical Practice

When the COVID-19 pandemic hit, education methods and learning experiences were abruptly changed, resulting in conflicts among students and educators. A more optimistic view of the COVID-19 contingency is that it created new opportunities for university education. Several factors were taken into consideration when these improvements were implemented, including nursing education. By enhancing students’ SDL skills, nursing educators were able to foster students’ creativity, interaction, and innovative learning as they transitioned from the traditional in-class learning methods to self-directed learning. Moreover, curricula and academic policies should undergo annual reviews and development. This encourages students to augment their autonomy in learning by directing them to acquire knowledge in a relevant and meaningful manner. Further, students’ awareness of their own SDL skills can be developed and enhanced through a variety of mechanisms, including the employment of learning contracts, promoting creative, innovative, critical thinking, and independent learning approaches, implementing contemporary teaching and assessment strategies that encourage SDL, and providing the necessary administrative and technical support systems.

## Figures and Tables

**Table 1:** Demographic characteristics of the study sample (*n* = 277).

Item	Number (%)	
Gender		
Male	119 (43)	
Female	158 (57)	
Age	Mean	20.51 ± 1.6
Marital Status		
Single	273 (98.6)	
Married	4 (1.4)	
Academic level		
Level 3	39 (14.1)	
Level 4	45(16.2)	
Level 5	30 (10.8)	
Level 6	81(29.2)	
Level 7	65 (23.5)	
Level 8	17 (6.1)	
Residence		
Riyadh	255 (92.4)	
Outside Riyadh	20 (7.2)	

**Table 2:** Results of Locus of Control subscale among the study sample.

Locus of Control Subscales	Minimum	Maximum	Mean (SD)
Personal control	18	90	56.38 (14.47)
Belief in chance	11	55	32.21 (7.97)
Meaningless of the effortfulness	10	50	29.37 (7.61)
Belief in fate	3	15	9.87 (2.61)
Belief in unjust world	5	25	14.76 (3.79)
Internal locus of control	18	90	56.37 (1.45)
External locus of control	29	235	86.2 (19.7)

**Table 3:** Self-directed learning among the study participants (*n* = 277).

SDL Subscales	Minimum	Maximum	Mean (SD)	Level # (%)	
Self-management	13	65	39.52(9.04)	Low	227 (81.9)
				High	50 (18.1)
Desire for learning	12	60	45.02 (10.38)	Low	121 (43.6)
				High	156 (56.4)
Self-control	15	75	52.36 (12.45)	Low	157 (56.7)
				High	120 (43.3)
Total level of readiness for SDL	40	200	144.0 (0.49)	Low	166 (59.9)
				High	111 (40.1)

# High level of readiness > 150, Low level of readiness < 150

**Table 4:** Correlation analysis of the LOC subscales and SDL readiness subscales.

SDL Readiness Subscales	Locus of Control Subscales					
	Personal Control	Belief in Chance	Meaninglessness of the Effortfulness	Belief in Fate	Belief in Unjust World	Overall LoC
Self-management	r = 0.38 * p = (0.0001)	r = 0.26 * p = (0.0001)	r = 0.25 * p = (0.0001)	r = 0.37 * p = (0.0001)	r = 0.33 * p = (0.0001)	X
Desire for learning	r = 0.33 * p = (0.0001)	r = 0.20 * p = (0.003)	r = 0.21 * p = (0.002)	r = 0.36 * p = (0.0001)	r = 0.24 * p = (0.001)	X
Self-control	r = 0.40 * p = (0.0001)	r = 0.24 * p = (0.001)	r = 0.22 * p = (0.001)	r = 0.39 * p = (0.0001)	r = 0.26 * p = (0.0001)	X
Overall Level of readiness for self-directed learning	Internal LOC	External LOC				X
	r = 0.22 * p = 0.0001	r = 0.10 *p* = 0.08				r = 0.19 * p = 0.001

* *p* ≤ 0.05.

## Data Availability

Data are available upon reasonable request.

## Public Involvement Statement

No public involvement in any aspect of this research.

## Guidelines and Standards Statement

This manuscript was drafted against the (STROBE) for a cross-sectional study.
